# Long-term outcomes of selective mutism: a systematic literature review

**DOI:** 10.1186/s12888-023-05279-6

**Published:** 2023-10-24

**Authors:** Miina Koskela, Tiia Ståhlberg, Wan Mohd Azam Wan Mohd Yunus, Andre Sourander

**Affiliations:** 1 Research Centre for Child Psychiatry, Institute of Clinical Medicine, Faculty of Medicine, University of Turku, Turku, Finland; 2 INVEST Research Flagship Center, University of Turku, Turku, Finland; 3 Department of Child Neurology, Turku University Hospital, Varha, Turku, Finland; 4 Department of Adolescent Psychiatry, Turku University Hospital, Varha, Turku, Finland; 5 Faculty of Social Sciences and Humanities, Universiti Teknologi Malaysia, Johor Bahru, Malaysia; 6 Department of Child Psychiatry, Turku University Hospital, Varha, Turku, Finland

**Keywords:** Anxiety disorder, Selective mutism, Long-term outcomes, Systematic literature review

## Abstract

**Background:**

Selective mutism (SM) is a childhood onset anxiety disorder, and the main symptom is not speaking in certain social situations. Knowledge about the duration and long-term outcomes of SM have been lacking and the aim of this systematic literature review was to address this gap in the literature. We investigated how long SM symptoms persisted as well as other psychiatric outcomes associated with SM in later life.

**Methods:**

The PubMed, PsycInfo, Web of Science, Cochrane Library and Embase databases were initially searched from inception to 11 September 2023. Studies were included if they were published in English and had followed up subjects with clinically diagnosed SM for at least two years. The review followed the Preferred Reporting Items of Systematic Reviews and Meta-analyses guidelines and the protocol was registered with the Open Science Framework. The papers were assessed using the Quality Assessment with Diverse Studies tool.

**Results:**

This review screened 2,432 papers and assessed 18 studies. Seven case series studies were excluded from discussion because of the low number of subjects and the fact that their findings could not be generalized to wider populations. In the end, nine clinical cohorts and two case control studies were reviewed. These provided a total of 292 subjects and the sample sizes ranged from 11–49. The overall quality of the studies was moderate. The review found that 190 of the 243 subjects in the studies that reported recovery rates showed moderate or total improvement from SM during follow up. Other anxiety disorders were the most common psychiatric disorders later in life, although these results should be interpreted with caution. Older age at baseline and parental psychopathology might predict greater impairment, but further studies are needed to confirm these results.

**Conclusions:**

Most subjects with SM recovered from this disorder during adolescence, but anxiety disorders were common in later life. Early detection and treatment are needed to prevent symptoms from persisting and other psychiatric disorders from developing.

**Supplementary Information:**

The online version contains supplementary material available at 10.1186/s12888-023-05279-6.

## Background

Selective mutism (SM) is an anxiety disorder that starts in childhood. The main symptom is that the children are unable to speak in certain situations, for example at school, despite speaking normally in other settings. The term is used in the Diagnostic and Statistical Manual of Mental Disorders, Fourth and Fifth Edition (DSM-IV and DSM-V). It is also used in the latest International Classification of Diseases, Eleventh Revision (ICD-11) and has replaced the less common term, elective mutism, which is used in the Tenth Revision (ICD-10). [1–5] The etiology of SM is still somewhat unknown, but it is likely that it comprises genetic, environmental and neurodevelopmental factors [6]. The prevalence of SM is quite low, at 0.18–1.9% [6, 7]. It has been reported to be slightly higher, at 2.2%, in immigrant populations [8].

SM often occurs together with other psychiatric and neurodevelopmental disorders. One register study on SM found that 69% of the subjects had additional psychiatric diagnoses and the most common ones were learning, affective, anxiety and childhood onset emotional disorders [9]. Other anxiety disorders are commonly comorbid with SM. A meta-analysis found that 80% of subjects with SM also had other anxiety disorders. The most common diagnoses were social phobia and elevated rates of specific phobias and separation anxiety disorder and generalized anxiety disorder were also observed [10]. Some studies have also found that SM was sometimes related to neurodevelopmental disorders [11, 12] or learning disorders [13, 14]. ICD-10 specified that SM could not be diagnosed with autism spectrum disorders (ASD) [1] and both DSM-V and ICD-11 advise caution when doing so [3, 4]. Despite that, there is some evidence about the shared temperamental features of these two disorders [15].

The current understanding is that SM symptoms persist throughout life to some extent [16, 17], but most people with childhood SM do not fulfil the diagnostic criteria by the time they reach late adolescence or adulthood. [18]. There have not been any systematic reviews on the long-term outcomes of SM, but there have been some systematic reviews about the short-term treatment outcomes [19–21]. A recent meta-analysis on the non-pharmacological treatment of SM reported promising results when behavioral interventions were used, but it also showed an urgent need for future research on treating SM. The authors did not evaluate the long-term outcomes of SM [22]. We are not aware of any studies on SM and suicidality. SM is often comorbid with social anxiety disorders (SAD) [10] and it has even been suggested that SM could be an extreme form of SAD [6, 15, 23, 24]. A systematic review on the long-term outcomes of SAD found that it was common for clinical subjects with SAD to have chronic symptoms [25]. It has not been confirmed if this also applies to SM.

Investigating the long-term outcomes of SM is important because it could help clinicians to plan the length of follow-up visits and treatment. It could also have implications for researchers who want to develop interventions to prevent future problems. The aim of this review was to address the lack of evidence on the long-term outcomes of SM by performing a systematic literature review that identified all the long-term follow-up studies that explored the psychiatric outcomes of SM. It aimed to examine the chronicity, rate of psychiatric comorbidity and suicidality of SM later in life. This review also reports if these studies used any kind of treatment and whether they identified factors that predicted the duration or severity of the SM symptoms.

## Methods

This systematic review was planned and conducted in line with the Preferred Reporting Items of Systematic Reviews and Meta-analyses (PRISMA) guidelines [26]. The study protocol was registered with Open Science on 12 May 2022 and updated on 28 October 2022 [27].

### Search strategy

An information specialist from the university library helped us to create, and finalize, the search terms and “selective mutism*” and “elective mutism*”, as used in ICD-10 and 11, and DSM-III, IV and V, were chosen. These were considered sensitive enough to capture all the relevant papers on the subject, while keeping the search manageable, as research on SM is scarce. The theory behind this strategy was that one of these two terms would be found in any paper that included subjects diagnosed with SM. These search terms were also used in the meta-analysis by Driessen et al. [10]. The PubMed, PsycInfo, Web of Science, Cochrane Library and Embase databases were searched from their inception until 31 May 2022 and the search was updated on 11 September 2023. In addition, the reference lists of the papers that were included in the full text review were checked for any other relevant papers. The university’s librarian provided access to the full texts that were not directly available through the electronic databases.

### Inclusion and exclusion criteria

The inclusion criteria were research studies or papers of any study design that had a minimum of two study subjects with clinically diagnosed SM and a follow-up period of at least two years from diagnosis. This time limit was chosen to exclude short-term treatment studies and because two years had been used by previous systematic literatures reviews on other anxiety disorders, such as social phobia [25]. The review included psychiatric disorders that were assessed at follow-up using any assessment methods, such as clinical evaluations or assessment forms. The exclusion criteria were unclear diagnostic methods or timing, unclear follow-up time and full texts not being available in English. Editorials, comments, letters, conference abstracts and reviews with no original data were also excluded.

### Study selection procedures

The results of the search were exported from each database to the Zotero reference manager (Roy Rosenzweig Center for History and New Media, Virginia, USA). The duplicates were removed using the reference manager tool. Two reviewers (MK, TS) screened the titles and abstracts independently, and all the relevant papers were included in the full text review. Any disagreements between the reviewers were resolved by discussion and consultation with the third author (WY).

### Quality assessment

The quality assessment was also carried out independently by two reviewers (MK, TS), with the Quality Assessment with Diverse Studies tool [28]. This tool was appropriate, because the current review included several different types of studies, namely cohort studies, case–control studies, and case series., Some of the studies reported qualitative results, and some had quantitative findings. Each study was assessed using all 13 sections of this tool and graded from 0–3 or not applicable for the study. After this assessment, the reviewers cross-checked their scores and any disagreements were resolved by discussion and consultation with the third author (WY). The mean scores were then calculated for each item and the total score was calculated. This quality assessment method does not have any pre-designed cut-off points to rate the quality of studies, but the instructions are to consider and discuss the quality of each criteria point-by-point.

### Data extraction and synthesis

The data were extracted by the first author into a shared Excel spreadsheet, version 2301 (Microsoft Corp, Washington, USA) and verified by the second author. The extraction table included the study and author, year, journal, country, study design, sample size and age range at baseline. It also included how SM was diagnosed, the treatment that was provided, the age range at follow up and the duration of the follow-up period. The table also included the outcome and how it was diagnosed or measured, the results, the possible predictors for the outcome and any comments. The studies were divided into two groups, based on the two main outcomes for this review: studies where the symptoms of SM were an outcome and studies where both SM and other psychiatric disorders were an outcome. If the study did not include the percentage of patients who had recovered during the follow-up periods, it was calculated for this review by using the reported number of subjects that had this positive outcome. A simplified table was created for publication (Table 1). Tables and figures were created using either the 2301 versions of Excel or Microsoft Word (Microsoft Corp). The results are reported following the PRISMA criteria and checklist, version 2020, and these additional data are presented in Table S1 (available online).

**Table 1:** Full list and details of the cohort and case–control studies included in the systematic literature review

**Study *Country***	**Study design *Treatment provided, if any***	**Sample size *Age at baseline***	**Length of follow up**	**Setting and diagnostic methods for mutism**	**Outcom ** ***Methods used to measure outcomes***	**Results summary at follow up**	**Predictors for outcomes**
**Cohort studies**							
Arajärvi, 1965 *Finland *[29]	Clinical cohort *Psychosocial, in-patient treatment*	*n* = 12 *4–8 years*	1–10 years	Clinical	SM symptoms *Questionnaires filled in by parents and one case investigated by clinician. Cognitive assessment, projective tests*	11/12 spoke in school after treatment but one still had SM symptoms	
Dogru, 2023 *Turkey *[30]	Clinical cohort *Psychotherapy and/or pharmacotherapy*	*n* = 49 *5–13 years*	N/A Duration of SM was 2.22 ± 1.35 years	Clinical data from patient records verified by comparing these to DSM-5 symptoms of SM	SM symptoms *(K-SADS-PL), CGI*	Mean duration of SM was 2.2 years. Duration of SM did not differ between males and females	Children with severe SM symptoms had a longer duration of illness and higher rates of psychiatric comorbidity
Kamani & Monga, 2020 *Canada *[16]	Clinical cohort *Psychosocial/ pharmacological. 74% received CBT. 45% received medication for anxiety, mainly SSRIs*	*n* = 31 22 with SM and 9 with just social anxiety disorder *4–14 years*	2–6 years mean = 4.2 years	Clinical (from patient records). Anxiety Disorders Interview Schedule for DSM-IV	**SM and social anxiety disorder** *Clinical, parent interview with ADIS, CGAS, SMQ and SCARED tools*	2/31 only had SM, 11/31 only had social anxiety disorder, 9/31 had both and 9/31 had neither	No difference between the types of treatment, not even for CBT Older age at baseline might predict impairment and higher rate of anxiety at follow up
Lang et al., 2016 *Israel *[31]	Clinical cohort *SM focused CBT*	*n* = 24 *6.40* ± *3.06 years*	2.90 ± 3.23 years	Clinical (from patient records)	**SM and comorbid psychiatric disorders** *Clinical, structured interviews and ADIS-IV-L, CGI and SMQ*	The recovery rate for SM was 84.2%. A significant decrease was observed in the levels of social phobias and specific anxiety disorder. No statistically significant improvement in other comorbidities after the follow-up period	
Lowenstein, 1979 *UK *[32]	Clinical cohort *Psychosocial*	*n* = 21 *3–8 years*	7 years	Clinical	SM symptoms *Not clear*	13/21 spoke normally 6/21 had some symptoms left 2/21 had SM	
Oerbeck et al., 2018 *Norway *[33]	Clinical cohort *School-based CBT intervention*	*n* = 30 *3–9 years*	5 years	Clinical	**Psychiatric diagnoses** *Clinical assessment of the child and structured interviews from parents and teachers. SSQ, SMQ, ADIS-IV, K-SADS-PL, ILC*	21/30 in full remission 5/30 in partial remission 4/30 fulfilled diagnostic criteria for SM 7/30 children (23%) fulfilled criteria for social phobia, and 2/30 had separation anxiety disorder, 3/30 had specific phobia and 1/30 had enuresis nocturna	Older age, symptom severity at baseline and familial SM were significant negative predictors for the outcome
Remschmidt et al., 2001 *Germany *[17]	Clinical cohort *Psychosocial. In-patient treatment, family counselling*	*n* = 41 *8.7* ± *3.6 years*	12.0 ± 5.2 years	Clinical	**SM symptoms, psychopathology symptoms, family psychopathology** *From patient records and 31 patients were personally assessed using standardized assessments. Interview included the Marburg Symptom Checklist and MBI or BI*	16/41 cases (39%) in remission 12/41 (29%) remarkable improvement. 8/41 (20%) mild improvement. 5/41 symptomatology remained unchanged. 10% had dysphoric mood. 19% had depression. 48% had impulsivity. 42% had severe psychopathological disturbances	Worse outcome was predicted by depressive/dysphoric mood, SM in the family, psychiatric disorders in the family, deviant parenting style were predictors
Sluckin et al., 1991 *UK *[34]	Clinical cohort *Psychosocial. Individual behavioural therapy or* *school based programs*	*n* = 25 *4–8 years*	2–10 years	Not stated, but study carried out in clinical settings	SM symptoms *Follow-up questionnaires for parents and teachers and Rutter Rating Scale for teachers*	9/11 in the behavioural group improved, 5/14 in the standard program improved. (Difference in the groups was significant, *p* < .05)	Behavioural group treatment for better outcome. Familial psychopathology for worse outcome (*p* < .05)
Wergeland, 1979 *Norway *[35]	Clinical cohort *6 received individual psychotherapy, including 4 who had in-patient treatment* *5 did not receive treatment*	*n* = 11 *6–12 years*	8–16 years	Clinical	**SM symptoms and psychopathology** *Interviews with parents, patients, and siblings. If needed, information from schools, employers and hospitals were obtained*	11/11 in remission from selective mutism. Two of four children, who had received in-patient treatment, were diagnosed with a neurotic disorder and one of those four was diagnosed with a psychotic disorder at follow up	
**Case–control studies**							
Kolvin & Fundudis, 1981 *UK *[36]	Case–control *Not specified. Received best treatment available at the time mentioned*	*n* = 24 *6–8 years*	5–10 years	Clinical	SM symptoms *Not clear*	11/24 had improved: 3/24 (12.5%) markedly improved, 8/24 (33%) moderately improved and 13/24 (54%) slightly or not improved	More girls than boys in the improved group. Less parental personality problems in the improved group. No statistical analyses made for these findings due to lack of power
Steinhausen et al., 2006 *Switzerland *[18]	Case–control *Not specified*	*n *= 33 *8.5* ± *3.1 years*	Not reported but the mean age at follow up was 21.6 ± 3.3 years	Clinical	**SM symptoms and any DSM-IV psychiatric diagnoses** *Clinical interview, clinical global improvement scale of SM, and psychiatric assessment by using CAPI version of the Munich-International Diagnostic Interview at follow up*	All displayed some improvement. 6/33 (18.2%) were slightly improved, 8/33 (24.2%) were markedly improved and 19/33 (57.6%) were totally improved. Subjects with SM had significantly more phobic disorders (*p* < 0.001) than healthy controls, but no more than controls with anxiety disorders. 14/33 (42.4%) had phobic disorders. 19/33 (57.6%) had any psychiatric diagnosis. More diagnoses than in healthy controls (p = 0.005)	Severity of SM at outcome was not predicted by any tested factors. Comorbidity at outcome: any psychiatric disorder was significantly predicted by family history of taciturnity (*p* = .04). Phobic disorders significantly predicted by immigrant status (*p* = .02)

*Abbreviations*: *SM* Selective mutism, *CBT* Cognitive behavioural therapy, *SSRI* Selective serotonin receptor inhibitor, *CGI* The Clinical Global Impression Scale, *ADIS* Anxiety and Related Disorders Interview Schedule, *CGAS* Children’s Global Assessment Scale, *SMQ* Selective Mutism Questionnaire, *SCARED* Screen for Child Anxiety Related Disorders, *SSQ* The school speech questionnaire, *K-SADS-PL* Schedule for Affective Disorders and Schizophrenia for School Aged Children (6–18 Years), *ILC* The inventory of life quality in children and adolescents, *MBI* The Mannheim Biographic Inventory, *BI* Biographic Inventory, *CAPI* The computer-assisted personal interview

## Results

**Fig. 1:**
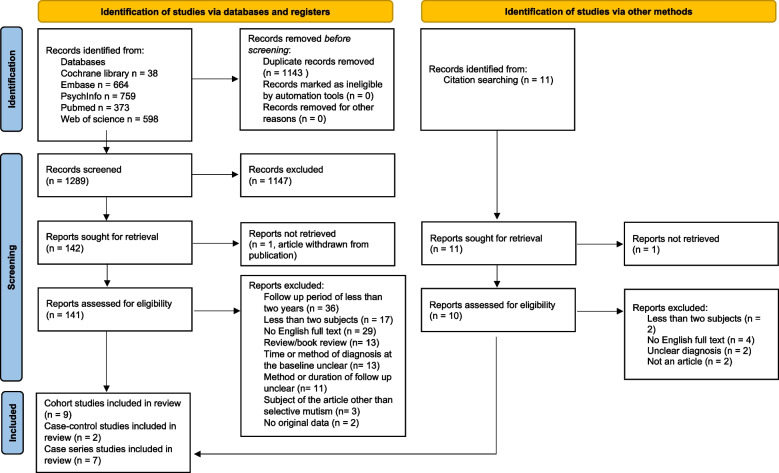
PRISMA 2020 flow diagram of the data selection process

The five databases yielded 2,200 titles during the initial search to 31 May 2022 and a further 232 titles during the second search up to 11 September 2023. There were 1,289 papers after any duplicates had been removed. After the titles and abstracts had been screened, 142 papers underwent a full text review. One of these had been withdrawn from publication and that left 141 papers. A further 123 papers were excluded for various reasons: the criteria for the follow-up time or diagnostic methods were not met, the paper was a review, it did not contain any original data, the paper was not in English, or the topic was not of interest. Table S2, (available online) provides further details. Another 11 papers were identified by checking the references of the included papers, but none of those were relevant when we screened the titles and abstracts. Three papers were excluded during the data extraction process, as they did not fulfil the inclusion criteria after closer evaluation, as the follow up periods or the timing of the SM diagnosis was unclear [37–39]. The final number of SM papers that were reviewed was 18. The search is described in Fig. 1.

The 18 studies that were included in the review were published between 1963 and 2022 and only seven papers had been published during the 10-year period to the search in September 2023. Eleven papers had been published since the turn of the century. Four of the studies came from the UK, four from Norway, two each from the USA and Turkey and there were single studies from Finland, Germany, Switzerland, Israel, Italy, and Canada. Nine of the studies were clinical cohorts that studied the outcomes of SM [16, 29–35] and two were case–control studies [18, 36]. Seven case series studies were excluded from the synthesis and discussion because of the low number of subjects and the fact that their findings could not be generalized to wider populations [40–46]. The results of these studies are not presented in the main tables, but they are briefly summarized in this paper and described in more detail in Table S3 (available online). Five of these studies were published after the year 2000 [40–42, 44, 46] and two of them were from the 1960s [43, 45]. The number of subjects varied from two [40, 41, 46] to five [44]. Six of these papers found that among all subjects the SM symptoms were lower at the follow-up than at baseline [40–43, 45, 46], but one paper found that three of the five subjects remained selectively mute throughout the study period [44].

The 11 case–control and cohort studies comprised a total of 292 subjects. The samples sizes of the cohort studies ranged from 11 to 49 subjects and the case control studies ranged from 24 to 33 subjects. The age at baseline ranged from three years [41] to 14 years [16]. The follow-up periods varied from two years [16] to 17 years [17]. Some of the subjects in one study were only followed up for a year, but the majority had been followed up for two years or more and the study was considered to meet the inclusion criteria for this review [29]. Five of the cohort and case control studies only examined SM symptoms at follow up and six studies examined SM and some other psychiatric symptoms or disorders. Four of the studies just used clinical interviews and did not report the use of any structured instruments, while seven used instruments or structured assessments to measure symptoms and reach diagnoses during the follow-up periods. The cohort and case–control studies, and the relevant data they provide, are listed in Table 1.

The methods and the quality of the studies varied. Five of the studies were published before year 2000, which may have had an impact on how comprehensive the articles were. The mean values were calculated for each of the quality assessment items in the case–control and cohort studies and these are presented in Table 2. The more detailed scores, including the values for the case series, are presented in Table S4 (available online). The case series were not included in the main results, as it was unclear how the cases were selected, and therefore the outcomes could have been biased. The general weaknesses of the studies were how they described the recruitment processes, the lack of data collection tools or poor justification for the data collection tools. There were also a number of studies that did not discuss the study limitations. None of the studies were excluded based on their quality. It should be noted that only two studies had control groups.

**Table 2:** Mean scores for the Quality Assessment with Diverse Studies items and the total score in cohort and case–control studies

Item	Score
Intro	2.5/3
Aims	2/3
Setting and target population	2/3
Design appropriate for the aims	2/3
Appropriate sampling	1.5/3
Rationale for data tools	2/3
Tool appropriate for aims	2/3
Data collection procedure	2/3
Recruitment data	2/3
Justification for analytic method	0.5/3
Method of analysis appropriate for aims	2/3
Evidence of stakeholder input	0/3
Strengths and limitations	2/3
**Total score**	23/39

The maximum total score ranged from 33–39, depending on which items were applicable for each study

### Results for mutism symptoms

**Fig. 2:**
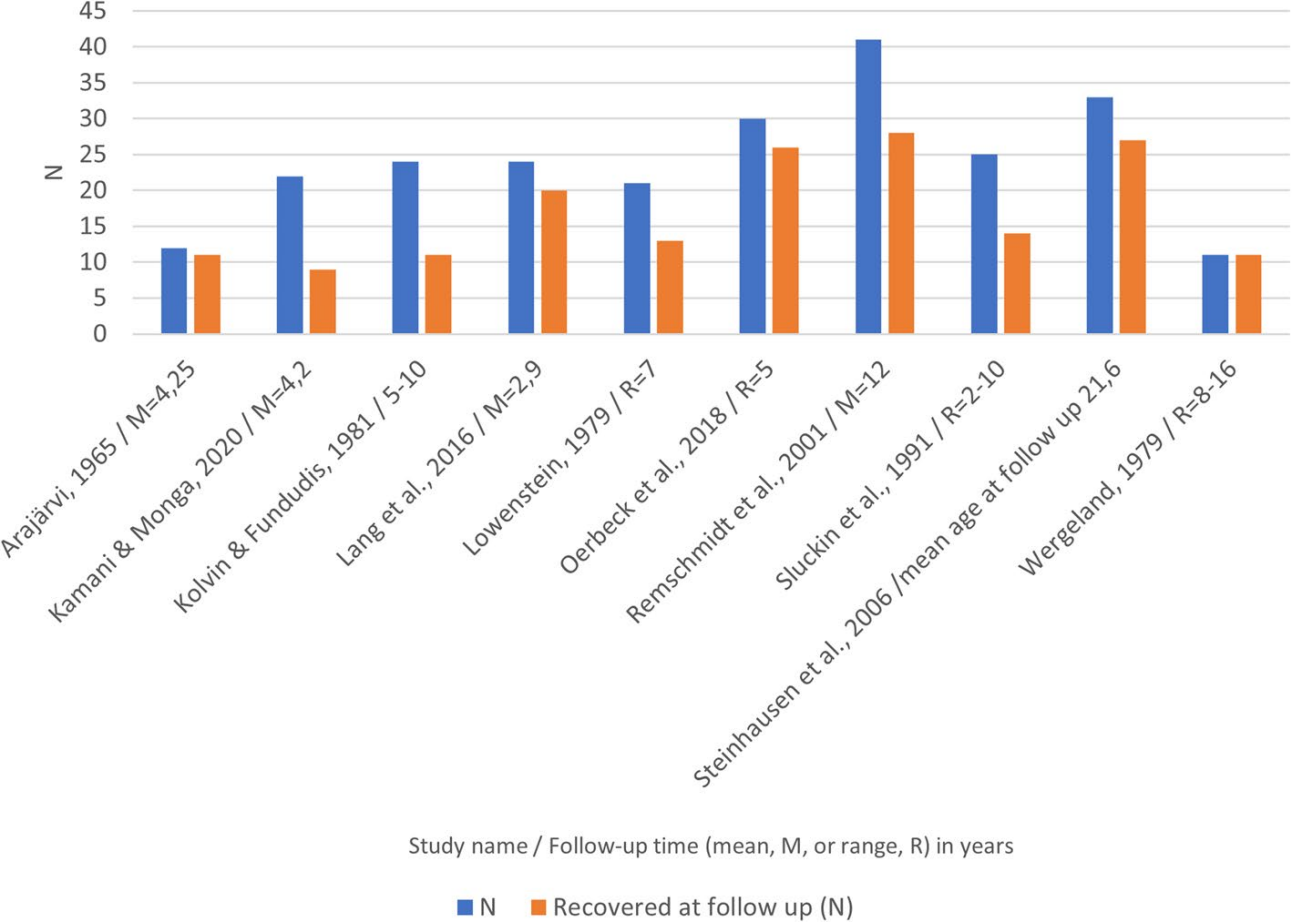
Subjects with SM that recovered from SM during the follow up periods in the cohort and case-control studies

The numbers of subjects who recovered from SM in the reviewed studies is presented in Fig. 2. The recovery rates in the cohort and case–control studies, which all had clinical based samples, ranged from 46% [36] to 100% [35]. The majority (190/243, 78%) of the subjects in these studies had shown moderate or total improvements in SM symptoms by the end of the follow-up periods. The study by Dogru did not report how many patients recovered from SM at a certain time point, but the authors did report that the duration of SM symptoms was 2.22 ± 1.35 years [30]. There were variations in how the results were reported. Some studies reported whether the subjects still fulfilled the diagnostic criteria of SM at follow up [16, 31], whereas others reported whether the subjects had shown mild, moderate or complete improvements [17, 18, 33, 36]. There were no clear associations between the follow-up time and how many subjects recovered from SM. The study by Kolvin followed up subjects for 5–10 years and only 11/24 recovered [36], whereas study by Lang had a mean follow-up time of 2.9 years and 20/24 recovered [31]. Three studies had a mean follow-up age that extended until early adulthood [17, 18, 35] and the number of subjects who showed total or moderate improvements in their SM symptoms varied from 68% [17] to 100% [35]. The paper by Kamani did not report how many subjects with SM had recovered from the disorder or how many had both SM and SAD as a baseline diagnosis [16]. The recovery rates from SM were good in the highest quality studies [17, 18, 33]. The paper by Remschmidt reported that the symptoms remained unchanged in 5/41 (12%) subjects, but said that the others had improved, at least slightly [17]. The study by Steinhausen reported that all subjects showed some improvement and 27/33 (81.8%) had improved markedly or completely [18]. The study by Oerbeck reported that only 4/30 (13.3%) still fulfilled the diagnostic criteria for SM after five years [33]. The studies that were published before 1980 reported recovery rates of almost 100%. However, the findings could have been biased by the different diagnostic criteria and methods used at that time [29, 35]. Most case series reported 100% recovery rates, but these cannot be generalized to the wider population, due to the low number of subjects and possible selection bias during recruitment [40–43, 45, 46].

*Sample sizes at the start (blue bars), sample sizes for those who recovered totally or moderately from SM symptoms (orange bars) and the median length of the follow-up periods (after each study).*

### Results for other psychiatric outcomes

**Fig. 3:**
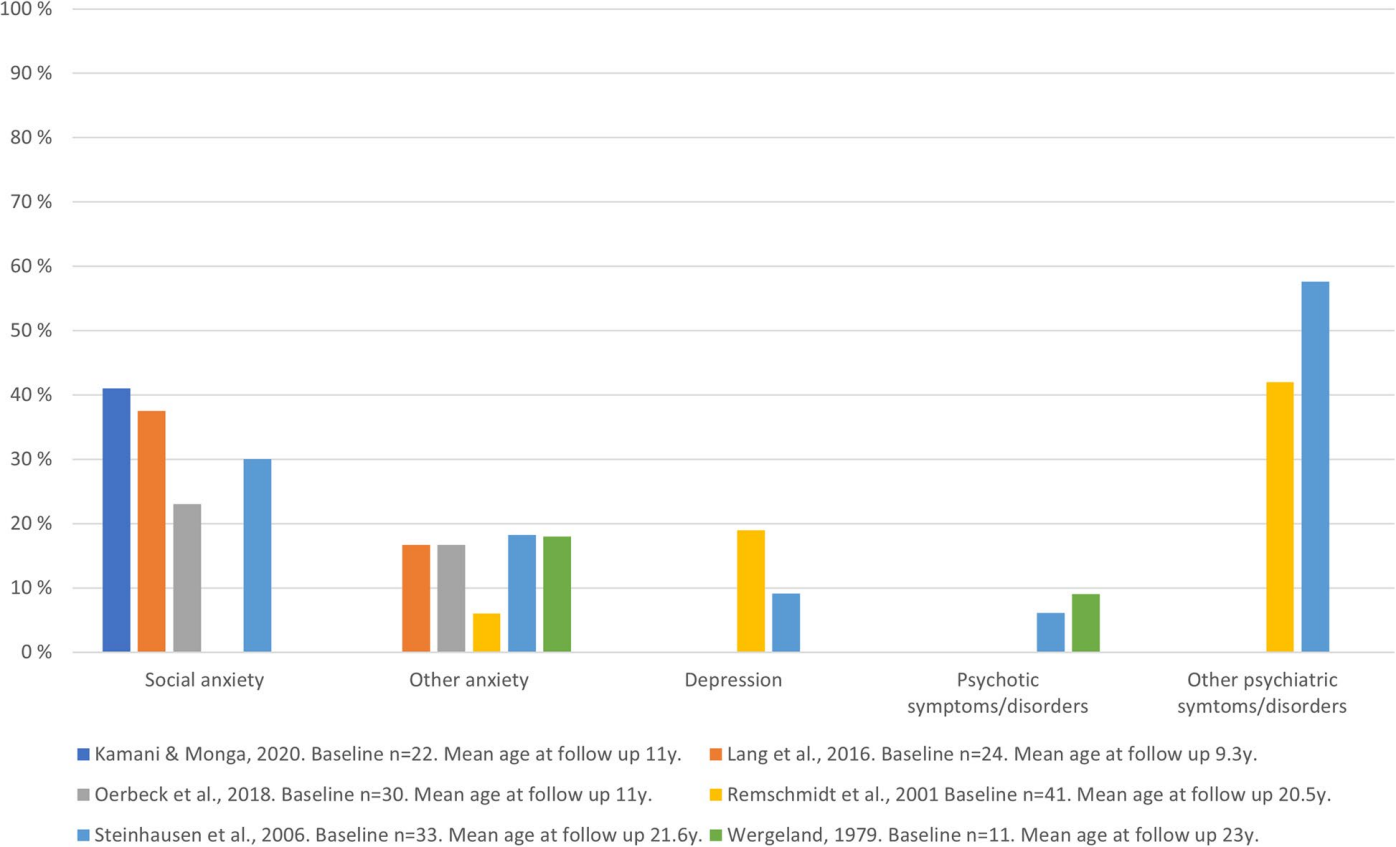
Percentages of the subjects with SM who presented with other psychiatric disorders at follow up from cohort and case-control studies

Five cohorts [16, 17, 31, 33, 35] and one case–control study [18] examined other psychiatric disorders at follow up and the results can be seen in Fig. 3. One study measured psychiatric symptoms as the outcomes [17], while other studies reported diagnoses based on clinical or diagnostic assessments. Each study reported how many subjects had a specific psychiatric problem or diagnosis at follow up. The observed outcomes comprised social anxiety disorder, other anxiety symptoms/disorders, depressive symptoms/disorders, psychotic symptoms/disorders, and other psychiatric symptoms/disorders.

The most common disorders at follow up in five of these six studies were anxiety disorders [16, 18, 31, 33, 35] and the levels ranged from 6% [17] to 54.2% [31]. The paper by Steinhausen found that subjects with SM had more phobic disorders and any psychiatric disorders than healthy controls. However, there were no statistically significant differences when these subjects were compared to controls with other anxiety disorders. [18] In addition, depression [17, 18] and psychotic symptoms [18, 35] were seen among the subjects with baseline SM. Two studies measured the overall rates of psychiatric disorders and these reported morbidity rates of 42% [17] and 58% [18]. Two studies compared the levels of other psychiatric disorders from baseline to the end of the follow-up and both found decreasing trends [17, 31]. One reported that general psychopathology decreased from 58 to 42% and the other found statistically significant decreases in the rates of social anxiety disorder (100 vs 37.5%) and specific phobia (45.8 vs 16.7%). Paper by Kamani studied 31 cases with SM or SAD, but did not clearly report which of the original SM cases had SM or social anxiety disorder at follow up [16]. At follow up, two subjects (6.5%) still had still SM, 11 (35.5%) only had SAD and nine (29.0%) had comorbid SM and SAD. [16] Study by Dogru reported, that 28 of their 39 subjects (57.1%) had psychiatric comorbidities, but did not state if the diagnoses were delivered, before, at the same time as or after the SM diagnoses [30]. No studies were found that reported suicidality among subjects with SM. Only three of the studies that were reviewed followed subjects into early adulthood [17, 18, 35]. These were also the only studies that reported other comorbid disorders, instead of just anxiety disorders. Only one study that reported comorbid disorders was published before the year 2000 [35].

### Treatment outcomes

The studies that were reviewed reported different methods for treating SM. It should be noted that this review focused on papers with a follow-up period of at least two years and this meant that the review did not include all the studies that examined treatment. Most of the subjects in the reviewed studies received some form of treatment. The limitations of the study designs prevented us from inferring that the treatment caused improvements among the cases. Several treatment methods were used by the studies: in-patient treatment, individual or group therapy, cognitive behavioral therapy (CBT), school interventions, changing schools or environments and selective serotonin reuptake inhibitors. The clinical cohorts reported that psychosocial treatment resulted in varying recovery rates from SM at the end of the follow-up periods. One study that used inpatient treatment reported a recovery rate of 92%, but the methods were not described [29]. A school-based CBT program achieved a recovery rate of 86% [33], the rate was 84% for an SM-focused CBT program [31] and it was 62% for therapy that used reinforcement and a familiar human as a catalyst [32]. An overall recovery rate of 56% was reported by a study that used individual behavioral treatment or school-based treatment. The recovery rate was higher in subjects that received behavioral treatment (82%) [34]. Another study reported a recovery rate of 39%, defined as full remission, for a program that used using various psychosocial treatments, parental counseling and, if needed, inpatient treatment [17]. In one study, the recovery rate at follow up was 50% for mixed treatment, meaning that some subjects received medication and some subjects received psychosocial interventions, with no differences between the treatment methods [16]. Study by Wergeland did not find statistically significant differences in the outcomes between six subjects who were treated and five who were not [35]. The case series presented good recovery rates from SM symptoms at follow up and all these studies offered some kind of treatment, detailed information in Table S3, available online.

### Prognostic factors

Some studies separately examined factors that were associated with outcomes, but very few had sufficient power to generate statistically significant findings. Factors that were associated with poorer outcomes were older age at baseline [33], symptom severity at baseline [33], depressive mood [17], familial SM [17, 33], familial psychopathology [17, 34], parental personality problems [36], the context of the muteness (not speaking at school, to strangers, to other children) [18] and male gender [36]. In addition, group treatment predicted better outcomes in one study [34].

Paper by Steinhausen studied predictors for SM severity and for other psychiatric disorders at follow up and none were associated with the severity of SM [18]. A family history of taciturnity, which means being reserved or reticent in conversation, was associated with any psychiatric disorder and immigrant status was associated with phobic disorders at follow up. When this study examined the symptomatic outcomes of SM, only the context in which the mutism occurred was significant. [18] Dogru et al. reported that children with severe SM symptoms were ill for a longer period of time and had higher rates of psychiatric comorbidity than children with moderate or mild symptoms [30].

## Discussion

This appears to be the first systematic literature review to explore long-term psychiatric outcomes of SM. It produced four main observations. First, most of the subjects with SM recovered from the disorder during the follow-up periods. Second, anxiety disorders were relatively common after the subject recovered from their SM symptoms. Third, the factors that might have predicted poor outcome were older age at the first diagnosis of SM and mutism and psychopathology in their immediate family. Fourth, the studies that were reviewed were small and lacked controls. No register-based studies were found and only two studies used case–control settings. Most studies were from western countries, especially Europe, which affected the representativeness of the results. Five of the studies were carried out before the publication of DSM-IV [29, 32, 34–36] and three of those even predated DSM-III [29, 32, 35]. This makes it difficult to be certain about the results of those studies because the diagnostic criteria have varied over the years. Only three studies continued to follow subjects until early adulthood [17, 18, 35].

The first finding was that most cases of mutism symptoms improved partly or completely over time. This was in line with current perceptions that SM usually occurs in childhood or early adolescence, even if symptoms persist to some extent. [6, 7] All the study subjects were clinical patients and most received some kind of treatment, which could partly explain the good recovery rates from SM seen in the studies. Some studies found that changing school was a key factor in the subject’s recovery [29, 35]. It might be that the SM symptoms persisted because of some environmental factors at their school and that changing school was an important factor in the subject’s recovery. It should be noted that four of the 11 cohort and case–control studies only measured recovery based on SM symptoms, but several studies suggested that other psychiatric and social problems were common, even after subjects recovered from their SM symptoms [16, 17, 47]. There was no clear association between recovery rates and the publication year of the studies.

Even though the recovery rates from SM were good in the reviewed studies, 22% of the subjects from the cohort and case–control studies had persistent SM symptoms [16–18, 29, 31–36]. Considering that the follow-up periods were fairly long, as they ranged from two to 17 years, that is a relatively large number of subjects who needed long-term services. Only three studies followed subjects until early adulthood and some subjects still suffered from SM symptoms as young adults [17, 18, 35]. One of those studies had significantly better outcomes [35], but it was carried out before DSM-III, which was first version of the Manual to publish diagnostic criteria for SM [10, 48]. A systematic literature review on five-year outcomes of SAD reported a 27% recovery rate for clinical samples and 40% for non-clinical samples, which was notably poorer than the recovery rate for SM in the current review [25]. This difference could be explained by the fact that SM develops into other anxiety disorders, especially social anxiety disorders, later in life [10, 16]. Even if a subject has recovered from SM, severe communication problems and social anxiety could continue.

The second finding was that five of the six cohort and case–control studies that examined other psychiatric disorders at follow up found moderate rates of anxiety disorders [16, 18, 31, 33, 35]. The follow-up period in these studies ranged from two years [16] to 16 years [35]. This finding was in line with a 2020 meta-analysis that reported that 80% of subjects with SM were diagnosed with a comorbid anxiety disorder [10]. Only four studies examined the prevalence of social phobia [16, 18, 31, 33] and the prevalence ranged from 23% [33] to 41% [16]. The association between SM and social phobia has been widely discussed [49–51] and this included whether SM is a symptom of a social phobia or its own disorder [52, 53]. The association between these two disorders could also be explained by the tendency for behavioral inhibition that was seen in both disorders [54]. Higher rates of social phobia were expected in the follow-up studies, but the relatively low rates could have been due to the small samples and unsystematic way of assessing psychiatric disorders. In addition, many of the studies were rather old and practices on how to diagnose these disorders with each other might have changed over time. For example, anxiety symptoms might have been considered to be part of SM, not its own disorder.

There were only a few findings for psychiatric outcomes other than anxiety. Depressive [17, 18] and psychotic [18, 35] symptoms and disorders were found only in a couple of studies, but this was probably because half of the studies only followed up subjects during childhood [16, 31, 33]. The age at onset for many psychiatric disorders is during adolescence or adulthood. Suicidality and self-harm behavior was not investigated by any of the studies. Anxiety disorders have generally been found to predict suicidal ideation and attempts [55]. Another diagnostic issue could have been misdiagnosis. SM could have been incorrectly diagnosed, especially if autism spectrum disorders and communication problems were present. A 2021 paper by Rødgaard reported that 1% of 2,199 children with autism had received a diagnosis of SM in childhood, compared to none of the 460,798 controls [56].

Most of the cases in the reviewed studies received some form of treatment, which could also explain the relatively good recovery rates from SM and the relatively low rates of comorbid psychiatric disorders during the follow-up periods. Previous meta-analyses found that psychosocial treatment was associated with better recovery rates from SM, compared to no treatment [20]. The level of anxiety and communication problems may have had an impact on recovery. A study by Tomohisa did not fulfill the criteria for this systematic literature review, because either the baseline or outcome diagnoses were not clinically diagnosed and the follow-up times were unclear [47]. However, the authors did report the factors that affected whether their subjects with self-reported SM felt that they had been cured of the disorder later in life. The study was an Internet survey of 77 subjects, aged 19–50 years, who had experienced SM during childhood, and it was noteworthy that the symptom levels were defined by retrospective self-reports. Just under half (48%) said that they felt they had been cured of SM and their retrospective SM symptom levels did not differ from those who did not feel they had been cured. However, those who did not feel they had been cured did say that they experienced more interpersonal anxiety [47].

The third finding was that older age at baseline, and mutism and other psychopathologies in the immediate family, might predict poorer SM outcomes. Risk factors for SM have been studied and the findings have included family psychopathology [9, 57]. Previous studies have showed that the parents of children with SM reported elevated rates of various disorders. These included increased rates of parental anxiety and social anxiety disorder [58–60] and other kinds of mental disorders, such as psychotic, mood and personality disorders [9]. Various different mental disorders among siblings have also been seen as a risk factor for SM, with the strongest associations between SM and childhood emotional disorders and ASD [61]. Other previously known risk factors for SM included speech and language problems in childhood [11, 62] and temperamental traits of behavioral inhibition [6]. Despite this, it remains unclear what factors affect poor outcomes of SM symptoms. Some of the reviewed studies investigated prognostic factors [16–18, 33, 34, 36], but unfortunately most of them did not have enough subjects to produce statistically significant findings. The findings of this review could imply that late discovery or late treatment onset could predict a more persistent course of symptoms, but further studies are needed to examine this association. A previous systematic literature review on SAD found that poorer outcomes were associated with comorbid personality disorders, higher symptom levels after treatment and the use of benzodiazepines after treatment [25].

The fourth finding of this review was that the quality of the reviewed papers was moderate. All the studies were based on clinical samples and there were no population-based studies or register studies. Many of the studies were descriptive case series, as nine out of the 18 studies did not contain any statistical analyses. This was reflected in the fact that only six of the 11 studies that were finally included scored more than 50% of the maximum quality score [16–18, 30, 31, 33, 34]. The diagnostic methods were poorly described in many studies and the methods for measuring outcomes differed, which made drawing conclusions complicated. Six studies used DSM-III or older diagnostic classifications, which could have biased the results, as those diagnostic criteria and standards differ from modern ones [17, 29, 32, 34–36]. Only three studies followed subjects until adulthood [17, 18, 35]. This means that the results on comorbid psychiatric disorders were probably biased, as the age at onset for a number of disorders is during adolescence or adulthood. The results should be interpreted with caution, as the quality of the studies was moderate or even poor and many lacked statistical analyses and modern diagnostic methods. The lack of published studies with high-quality designs limited firm conclusions and clinical implications about long-term symptomatic outcomes, rates of recovery and prevalence. The current review highlights the urgent need for larger, methodologically strong, long-term follow-up studies in the future.

There were other limitations in the current review that should also be considered. The review may not have picked up some studies due to their unclear reporting of diagnostic methods and follow-up periods. In addition, some studies were only published in other languages, mostly German, and this means that some studies with important information could have been missed. This review was also restricted to studies that included subjects with diagnosed SM, which might have excluded some surveys or school-based studies. It was not possible to conduct a meta-analysis of the selected studies, because only two had a case–control setting and many were descriptive in nature. Therefore, the results of the current review also remain descriptive. The aim of this study was restricted to searching for psychiatric outcomes, which means that it cannot answer the question about whether SM in childhood effects an individual’s quality of life in adulthood. The review did aim to assess whether there were high levels of suicidality among subjects with SM but was unable to find any papers that covered that subject and fulfill that aim.

## Conclusions

This review showed that, although some studies examined the long-term outcomes of SM, larger sample sizes, methodologically sound designs, valid diagnostic assessments, and long-term follow-up periods are warranted. The long-term recovery rates for SM were relatively good in the reviewed studies, but other disorders, mainly anxiety disorders, were common later in life. Early detection and treatment are needed to prevent symptoms persisting and other psychiatric disorders from developing. The wellbeing of a child’s family, and the family dynamics, should be kept in mind when treating SM, as many factors related to families were associated with poorer SM outcomes. Sometime, changing school helped a child to recover from SM and this could suggest that school factors, such as bullying, could cause SM symptoms to continue. In addition, long-term follow-up, and providing children with SM with more support to communicate, may play important roles in avoiding future problems.

### Supplementary Information

**Additional file 1: Table S1. **Prisma 2020 checklist. **Table S2.** Exclusion criteria in full text review First. **Table S3.** Description of case studies, that followed up subjects more than two years. **Table S4.** Quality Assessment by the Quality Assessment with Diverse Studies.

## Data Availability

All the material and tables in this paper are available in the paper or as additional tables.

## Abbreviations

SM: Selective mutism
ICD: International Classification of Diseases
DSM: Diagnostic and Statistical Manual of Mental Disorders
SAD: Social anxiety disorder
PRISMA: The Preferred Reporting Items of Systematic Reviews and Meta-analyses
CBT: Cognitive behavioral therapy
