# Supplementary material for: Long-term outcomes of selective mutism: a systematic literature review
Source: BMC Psychiatry. 2023 Oct 24;23:779. doi: 10.1186/s12888-023-05279-6 (PMC10598940; doi:10.1186/s12888-023-05279-6)
Supplement: Supplementary file 1 — Additional file 1: Table S1. Prisma 2020 checklist. Table S2. Exclusion criteria in full text review First. Table S3. Description of case studies, that followed up subjects more than two years. Table S4. Quality Assessment by the Quality Assessment with Diverse Studies. [file 12888_2023_5279_MOESM1_ESM.pdf]

Additional data

Long-term outcomes of Selective Mutism – Systematic literature review

BMC Psychiatry

Miina Koskela[1,2], Tiia Ståhlberg[1,2], Wan Mohd Azam Wan Mohd Yunus[1,2,4], Andre Sourander[1,2,3]

1. Research Centre for Child Psychiatry, Institute of Clinical Medicine, Faculty of Medicine, University of Turku, Turku, Finland
2. INVEST Research Flagship Center, University of Turku, Turku, Finland.
3. Department of Child Psychiatry, Turku University Hospital, Turku, Finland.
4. Faculty of Social Sciences and Humanities, Universiti Teknologi Malaysia, Malaysia

Corresponding author:

Miina Koskela

Email: miikosy@utu.fi

Table S1: Prisma 2020 checklist

Section and Topic	Item #	Checklist item	Location where item is reported
**TITLE**			
Title	1	Identify the report as a systematic review.	page 1
**ABSTRACT**			
Abstract	2	See the PRISMA 2020 for Abstracts checklist.	
**INTRODUCTION**			
Rationale	3	Describe the rationale for the review in the context of existing knowledge.	pages 2-3
Objectives	4	Provide an explicit statement of the objective(s) or question(s) the review addresses.	page 3
**METHODS**			
Eligibility criteria	5	Specify the inclusion and exclusion criteria for the review and how studies were grouped for the syntheses.	page 4
Information sources	6	Specify all databases, registers, websites, organisations, reference lists and other sources searched or consulted to identify studies. Specify the date when each source was last searched or consulted.	page 3
Search strategy	7	Present the full search strategies for all databases, registers and websites, including any filters and limits used.	page 3
Selection process	8	Specify the methods used to decide whether a study met the inclusion criteria of the review, including how many reviewers screened each record and each report retrieved, whether they worked independently, and if applicable, details of automation tools used in the process.	page 4
Data collection process	9	Specify the methods used to collect data from reports, including how many reviewers collected data from each report, whether they worked independently, any processes for obtaining or confirming data from study investigators, and if applicable, details of automation tools used in the process.	pages 4-5
Data items	10a	List and define all outcomes for which data were sought. Specify whether all results that were compatible with each outcome domain in each study were sought (e.g. for all measures, time points, analyses), and if not, the methods used to decide which results to collect.	pages 3-4
	10b	List and define all other variables for which data were sought (e.g. participant and intervention characteristics, funding sources). Describe any assumptions made about any missing or unclear information.	pages 4-5
Study risk of bias assessment	11	Specify the methods used to assess risk of bias in the included studies, including details of the tool(s) used, how many reviewers assessed each study and whether they worked independently, and if applicable, details of automation tools used in the process.	page 4
Effect measures	12	Specify for each outcome the effect measure(s) (e.g. risk ratio, mean difference) used in the synthesis or presentation of results.	N/A
Synthesis methods	13a	Describe the processes used to decide which studies were eligible for each synthesis (e.g. tabulating the study intervention characteristics and comparing against the planned groups for each synthesis (item #5)).	page 4
	13b	Describe any methods required to prepare the data for presentation or synthesis, such as handling of missing summary statistics, or data conversions.	N/A
	13c	Describe any methods used to tabulate or visually display results of individual studies and syntheses.	pages 4-5
	13d	Describe any methods used to synthesize results and provide a rationale for the choice(s). If meta-analysis was performed, describe the model(s), method(s) to identify the presence and extent of statistical heterogeneity, and software package(s) used.	pages 4-5

Section and Topic	Item #	Checklist item	Location where item is reported
	13e	Describe any methods used to explore possible causes of heterogeneity among study results (e.g. subgroup analysis, meta-regression).	N/A
	13f	Describe any sensitivity analyses conducted to assess robustness of the synthesized results.	N/A
Reporting bias assessment	14	Describe any methods used to assess risk of bias due to missing results in a synthesis (arising from reporting biases).	N/A
Certainty assessment	15	Describe any methods used to assess certainty (or confidence) in the body of evidence for an outcome.	N/A
**RESULTS**			
Study selection	16a	Describe the results of the search and selection process, from the number of records identified in the search to the number of studies included in the review, ideally using a flow diagram.	Fig1
	16b	Cite studies that might appear to meet the inclusion criteria, but which were excluded, and explain why they were excluded.	page 5
Study characteristics	17	Cite each included study and present its characteristics.	table 1
Risk of bias in studies	18	Present assessments of risk of bias for each included study.	table s2
Results of individual studies	19	For all outcomes, present, for each study: (a) summary statistics for each group (where appropriate) and (b) an effect estimate and its precision (e.g. confidence/credible interval), ideally using structured tables or plots.	fig 3 and 4
Results of syntheses	20a	For each synthesis, briefly summarise the characteristics and risk of bias among contributing studies.	pages 4-7
	20b	Present results of all statistical syntheses conducted. If meta-analysis was done, present for each the summary estimate and its precision (e.g. confidence/credible interval) and measures of statistical heterogeneity. If comparing groups, describe the direction of the effect.	N/A
	20c	Present results of all investigations of possible causes of heterogeneity among study results.	N/A
	20d	Present results of all sensitivity analyses conducted to assess the robustness of the synthesized results.	N/A
Reporting biases	21	Present assessments of risk of bias due to missing results (arising from reporting biases) for each synthesis assessed.	N/A
Certainty of evidence	22	Present assessments of certainty (or confidence) in the body of evidence for each outcome assessed.	N/A
**DISCUSSION**			
Discussion	23a	Provide a general interpretation of the results in the context of other evidence.	pages 9-11
	23b	Discuss any limitations of the evidence included in the review.	page 11
	23c	Discuss any limitations of the review processes used.	page 11
	23d	Discuss implications of the results for practice, policy, and future research.	page 11
**OTHER INFORMATION**			
Registration and protocol	24a	Provide registration information for the review, including register name and registration number, or state that the review was not registered.	page 3
	24b	Indicate where the review protocol can be accessed, or state that a protocol was not prepared.	page 3/references
	24c	Describe and explain any amendments to information provided at registration or in the protocol.	page 3

Section and Topic	Item #	Checklist item	Location where item is reported
Support	25	Describe sources of financial or non-financial support for the review, and the role of the funders or sponsors in the review.	page 11
Competing interests	26	Declare any competing interests of review authors.	page 12
Availability of data, code and other materials	27	Report which of the following are publicly available and where they can be found: template data collection forms; data extracted from included studies; data used for all analyses; analytic code; any other materials used in the review.	page 12

From: Page MJ, McKenzie JE, Bossuyt PM, Boutron I, Hoffmann TC, Mulrow CD, et al. The PRISMA 2020 statement: an updated guideline for reporting systematic reviews. BMJ 2021;372:n71. doi: 10.1136/bmj.n71

For more information, visit: http://www.prisma-statement.org/

Table S2: Exclusion criteria in full text review

First author and year	Title	Reason for exclusion
Ale et al., 2013	Two cases of early childhood selective mutism: Variations and treatment complexities	Follow-up period of less than two years
Ambrosino & Alessi, 1979	Elective mutism – Fixation and the double blind	Less than two subjects
Andersson & Thomssen, 1998	Electively mute children: An analysis of 37 Danish cases	Follow-up period of less than two years
Ashton, 1996	Selective mutism in children - Balderson,C	Book review
Atoynatan, 1986	Elective mutism – Involvement of the mother in the treatment of the child	Less than two subjects (only one case fulfilling the follow-up criteria)
Aubry & Espasa, 2003	Le mutisme sélectif: Étude de 30 cas = Selective muteness: A study of 30 cases	No English full text
Barterian et al., 2018	An Examination of Fluoxetine for the Treatment of Selective Mutism Using a Nonconcurrent Multiple-Baseline Single-Case Design Across 5 Cases	Follow-up period of less than two years
Barterian, 2015	Fluoxetine for the treatment of selective mutism with elevated social anxiety symptoms: A nonconcurrent multiple baseline design across five cases	Follow-up period of less than two years
Beck & Warnke, 2003	Day care treatment of two sibling with selective mutism	No English full text
Blum et al., 1998	Case study: Audio feedforward treatment of selective mutism	Follow-up period of less than two years
Boggs, 2005	Selective Mutism Anxiety Reduction Therapy: A multiple case study	Follow-up period of less than two years
Boneff-Peng, 2023	An Updated Characterization of Childhood Selective Mutism: Exploring Clinical Features, Treatment Utilization, and School Services.	Follow-up period of less than two years
Bozigar & Hansen, 1984	Group treatment of elective mute children	Follow-up period of less than two years
Brewer & Sarvet, 2011	Management of anxiety disorders in the pediatric primary care setting	Review
Calhoun & Koenig, 1973	Classroom modification of elective mutism	Follow-up period of less than two years
Chavira et al., 2004	Child anxiety in primary care: Prevalent but untreated	Follow-up period of less than two years
Cohan et al., 2008	Refining the classification of children with selective mutism: A latent profile analysis	No follow-up period
Cornacchio, 2019	Evaluating intensive group behavioral treatment for children with selective mutism	Follow-up period of less than two years
Crundwell, 2006	Identifying and Teaching Children With Selective Mutism	Review
Cupalova & Novotny, 1981	Child mutism from the point of view of the physician and the teacher	No English full text
Denis et al., 2013	Mutism	No English full text
Eggers, 1995	Selective mutism in children – Cline, T., Baldwin, S	Book review
Ehrsam & Heese, 1956	Pädagogische Betrachtungen zum elektiven Mutismus = Pedagogical considerations in elective mutism	No English full text

Éva, 2004	A szelektív mutizmus kezelésének lehetőségei = Possibilities of treatment of selective mutism	No English full text
Fung et al., 2002	Web-based CBT for selective mutism	Less than two subjects
Golwyn & Sevlie, 1999	Phenelzine treatment of selective mutism in four prepubertal children	Time or method of diagnosis at baseline unclear
Gray & Livingston, 2001	Two sets of twins with selective mutism: a case presentation	Time or method of diagnosis at baseline unclear
Gross & Bernstein, 1981	Treating elective mutism using stimulus compounding and reinforcement	Less than two subjects
Grosso et al., 1999	Selective mutism, speech delay, dysmorphisms, and deletion of the short arm of chromosome 18: A distinct entity?	Less than two subjects
Hartmann, 2004	Mutismus in der Schule - Ein unlösbares Problem? = Mutism at school - An unsolvable problem?	No English full text
Harvey & Milne, 1998	Pharmacotherapy of selective mutism: Two case studies of severe entrenched mutism responsive to adjunctive treatment with fluoxetine	Time or method of diagnosis at baseline unclear
Heimlich, 1981	Patient as assistant therapist in paraverbal therapy with children	Follow-up period of less than two years
Holka-Pokorska et al., 2018	The controversy around the diagnosis of selective mutism - A critical analysis of three cases in the light of modern research and diagnostic criteria	Time or method of diagnosis at baseline unclear
Hu, 2022	Selective mutism in China: a nationwide survey and case-control study	Follow-up period of time unclear
Hudson et al., 2001	Expanding horizons: Adapting manual-based treatments for anxious children with comorbid diagnoses	Follow-up period of less than two years / less than two subjects
Jones & Odell-Miller, 2022	A theoretical framework for the use of music therapy in the treatment of selective mutism in young children: Multiple case study research	Follow-up period of less than two years
Kee et al., 2001	An electronic communication device for selective mutism	Less than two subjects
Kehle et al., 2012	Augmented self-modeling as an intervention for selective mutism	Less than two subjects (only one case fulfilling the follow up criteria)
Kehle et al., 1998	Augmented self-modeling as a treatment for children with selective mutism	Follow-up period of less than two years
Keville, 2023	Parent perspectives of children with selective mutism and co-occurring autism	Follow-up period of less than two years
Klin & Volkmar, 1993	Elective mutism and mental retardation	Time or method of diagnosis at baseline unclear
Kristensen, 2003	The selective mutism resource manual	Book review
Krohn et al., 1992	A study of the effectiveness of a specific treatment for elective mutism	Follow-up period of time unclear
Krolian, 1988	Speech is silvern, but silence is golden': Day hospital treatment of two electively mute children	Follow-up period of time unclear
Kupietz & Schwartz, 1982	Elective mutism: evaluation and behavioral treatment of three cases.	Follow-up period of less than two years
Kuhl, 1977	The symbiotic deficit and speechlessness	No English full text
Kurth & Schweigert, 1972	Causes and courses of mutism in children	No English full text

Lorand, 1960	Follow-up period of of children with elective mutism.	No English full text
Lowenstein & Lowenstein, 1976	The treatment and follow-up of a number of cases of elective mutism	Follow-up period of time unclear
MacGregor et al., 1994	Silent at school - Elective mutism and abuse	Follow-up period of less than two years
Mackenberg, 1998	Understanding children who refuse to speak - Communication and coping in elective mutism	No English full text
Marhoon et al., 2002	Child psychiatric characteristics and course of elective mutism: An analysis of 40 cases from Bahrain	Follow-up period of time unclear
Matson et al., 1992	Treatment of elective mute behavior in two developmentally delayed children using modeling and contingency management	Follow-up period of less than two years
Matson, 1983	Selective mutism – Implications for research and treatment – Kratockwill, T	Book review
Matsumura, 1992	An application of fading procedure in the school to facilitate generalization of appropriate speech of electively mute children	No English full text
Melfsen et al., 2021	Selective mutism: The "unsafe world model"	No follow-up period
Melfsen & Walitza, 2017	Therapy of Selective Mutism	No English full text
Misch, 1952	Elektiver Mutismus im Kindesalter = Elective mutism in childhood	No English full text
Mitchell & Kratochwill, 2013	Treatment of selective mutism: Applications in the clinic and school through conjoint consultation	Follow-up period of less than two years
Molina et al., 1999	Mutismo selectivo = Selective mutism	No English full text
Money, 1968	Psychologic approach to psychosexual misidentity with elective mutism: sex reassignment in two cases of hyperadrenocortical hermaphroditism	Not a follow-up study for mutism
Monzo et al., 2015	The mutism of the mind: child and family therapists at work with children and families suffering with selective mutism	Time or method of diagnosis at baseline unclear
Moos, 1941	Über elektiven Mutismus bei Kindern = Elective mutism in children	No English full text
Mora et al., 1962	Dynamics and psychotherapy of identical twins with elective mutism	Time or method of diagnosis at baseline unclear
Moreno & Predreira, 1998	El tratamiento del mutismo electivo: Integración de psicoterapia y fluoxetina = Treatment of elective mutism in children with fluoxetine	No English full text
Morris, 1953	Cases of elective mutism	Follow-up period of time unclear
Motavalli, 1995	Fluoxetine for (s)elective mutism [2]	Less than two subjects
Mullen, 2015	Tackling selective mutism: a guide for professionals and parents	Book review
Muris, 2023	Symptoms of Selective Mutism in Middle Childhood: Psychopathological and Temperament Correlates in Non-clinical and Clinically Referred 6- to 12-year-old Children.	Follow-up period of less than two years
Najeeb, 2023	A Case Study of Depression in High Achieving Students Associated With Moral Incongruence, Spiritual Distress, and Feelings of Guilt.	Follow-up period of less than two years
Nash et al., 1979	Management program for elective mutism	Time or method of diagnosis at baseline unclear
Nesnidalová, 1957	Elective mutism in child psychiatric practice	No English full text
Nyström, 1973	[4 cases of elective mutism].	No English full text

Oerbeck et al., 2015	Selective mutism: follow-up study 1 year after end of treatment	Follow-up period of less than two years
Oerbeck et al., 2014	A randomized controlled trial of a home and school-based intervention for selective mutism - defocused communication and behavioural techniques	Follow-up period of less than two years
Ohi et al., 1982	A clinical and psychopathological consideration on elective mutism in adolescence: five cases who have poor volition to seek socialization (author's transl)	No English full text
Ohi et al., 1979	A consideration on elective mutism in childhood	No English full text
Omdal, 2015	Tackling selective mutism: a guide for professionals and parents	Book review
Omdal, 2007	Can adults who have recovered from selective mutism in childhood and adolescence tell us anything about the nature of the condition and/or recovery from it?	Time or method of diagnosis at baseline unclear
Ooi et al., 2012	Application of a web-based cognitive-behavioural therapy programme for the treatment of selective mutism in Singapore: A case series study	Follow-up period of less than two years
Park et al., 2007	Clinical case rounds in child and adolescent psychiatry: Selective mutism	Less than two subjects
Paul, 2011	Helping Children with Selective Mutism: A Guide for School-Based Professionals	Book review
Perçinel & Yazici, 2014	Okul öncesi dönem selektif mutizm vakalarinda fluoksetin tedavisi: Iki olgu eşliğinde tartişma = Fluoxetine treatment in preschool cases with selective mutism: Discussion through two cases	No English full text
Pereira et al., 2019	Speech problems and speech delay: Possible underdiagnosis of selective mutism	Less than two subjects
Porjes, 1992	Intervention with the selectively mute child	Follow-up period of time unclear
Pustrom & Speers, 1964	Elective mutism in children	Follow-up period of time unclear
Richards & Hansen, 1978	A further demonstration of the efficacy of stimulus fading treatment of elective mutism	Less than two subjects
Roe, 1993	An interactive therapy group	Follow-up period of less than two years
Russell et al., 1998	Multimodal intervention for selective mutism in mentally retarded children	Follow-up period of less than two years
Rødgaard et al., 2021	Childhood diagnoses in individuals identified as autistics in adulthood	Not a follow-up study for mutism
Sanok & Striefel, 1979	Elective mutism – generalization of verbal responding across people and settings	Less than two subjects
Schieveld & Sallin, 2021	Pervasive refusal syndrome revisited: a conative disorder	Not a follow-up study for mutism
Schmidtke, 1978	Model learning as behavior-therapeutic strategy in two electively mute girls	No English full text
Schmidtke & Schaller, 1978	[Learning on a model as behavior-therapeutic strategy in elective mutism].	No English full text
Schwartz et al., 2006	Selective mutism: Are primary care physicians missing the silence?	Follow-up period of time unclear
Schwenck & Gensthaler, 2017	Psychopathology of Selective Mutism	No English full text
Segal, 1999	Silent partners: twins with selective mutism.	Review
Segal, 2006	Female monozygotic twins with selective mutism--a case report.	No original data

Segal, 2003	Insightful contribution to empirical analyses of selective mutism (SM).	No original data
Shorer et al., 2022	The Integrated Behavior Therapy for Children with Selective Mutism: Findings from an open pilot study in a naturalistic setting	Follow-up period of less than two years
Shvarztman et al., 1990	Elective mutism in family practice	Time or method of diagnosis at baseline unclear
Silverman & Powers, 1970	Elective mutism in childhood	Time or method of diagnosis at baseline unclear
Simons et al., 1997	Elective mutism and chromosome 18 abnormality	Less than two subjects
Sluckin, 1977	Children who do not talk at school	Follow-up period of less than two years
Steffenburg et al., 2018	Children with autism spectrum disorders and selective mutism	No follow-up period
Stein, 2023	Are There Reasons to Fear Anxiety Screening?	Review
Szabo, 1996	Selective mutism and social anxiety.	Less than two subjects
Tachibana et al., 1982	Elective mutism in identical twins	No English full text
Teece, 2004	What selective mutism means to us	Paper withdrawn
Toma & Toyama, 2021	Adaptive and maladaptive processes in individuals who had experienced selective mutism	No English full text
Tomohisa, 2022	Long-term outcome of selective mutism: factors influencing the feeling of being cured.	Follow-up period of time unclear
Tramer, 1934	Elektiver Mutismus bei Kindern = Selective mutism of children	No English full text
Turkiewicz et al., 2008	Selective mutism and the anxiety spectrum - A long-term case report	Less than two subjects
Vecchio & Kearney, 2009	Treating Youths With Selective Mutism With an Alternating Design of Exposure-Based Practice and Contingency Management	Follow-up period of less than two years
Wang, 2023	Practical Tips for Paediatricians: Helping kids find their voices in selective mutism	Review
Weber, 1950	Zum elektiven Mutismus der Kinder = Elective mutism in children	No English full text
Weininger, 1987	Electively mute children: A therapeutic approach	Follow-up period of time unclear
Weinstock et al., 2020	Behavioral Assessment and Treatment of Selective Mutism in Identical Twins	Time or method of diagnosis at baseline unclear
Wilkins, 1985	A comparison of elective mutism and emotional disorders in children	Time or method of diagnosis at baseline unclear
Williamson et al., 1977	Behavioural treatment of elective mutism – 2 case studies	Follow-up period of less than two years
Wright et al., 1985	Early identification and intervention with children who refuse to speak	Follow-up period of less than two years
Wulbert et al., 1973	Efficacy of stimulus fading and contingency management in treatment of elective mutism – case study	Less than two subjects
Zhang, 2023	Demographic and clinical profiles of preschool children with selective mutism in Singapore.	Follow-up period of less than two years
Zanni, 2014	Anxiety disorders: Real disease, real treatment	Review

Clinical Commentary, 2014	Clinical material	Less than two subjects
**In addition, following articles were found through reference check but all were excluded based on the abstract. Reasons as below.**		
Funke et al. 1978	Klinische Untersuchungen und Therapie bei Kindern mit selektivem Mutismus.	No English full text
Hayden, 1980	The classification of elective mutism.	Time or method of diagnosis at baseline unclear
Heil et al. 1985	Home treatment.	Less than two subjects
Kolvin, 1994	The origin of elective mutism.	Congress abstract
Mitchell, 1985	Long standing elective mutism.	Not found
Murray, 1983	A stubborn silence.	Less than two subjects
Poller, 1990	Mutismus bei Kindern und Jugendlichen. Eine katamnestische Untersuchung.	No English full text
Rutter, 1977	Speech delay.	Book chapter
Rösler, 1981	Befunde beim neurotischen Mutismus der Kinder. Eine Untersuchung an 32 mutistischen Kindern.	No English full text
Schachter, 1977	Le mutisme electif chez l'cnfant d'age prescolaire et scolaire: Contribution a la psychopathologie de la communication	No English full text
Wright, 1968	A clinical study of children who refuse to talk in school.	Time or method of diagnosis at baseline unclear

Table S3: Description of case studies, that followed up subjects more than two years

**Study**<br><br>***Country***	**Study design**<br><br>***Treatment provided, if any***	**Sample size**<br><br>***Age at baseline***	**Length of follow up**	**Setting and diagnostic methods for mutism**	**Outcome**<br><br>***Methods used to measure outcomes***	**Results summary at follow up.**	**Predictors for outcomes**
Albrigtsen et al., 2016<br><br>*Norway*	Case series<br><br>*Psychosocial and pharmacological (fluoxetine), in-patient treatment.*	n=2<br><br>*7 years*	7 years	clinical	SM symptoms<br><br>*Not clear*	At the age of 13, all cases spoke spontaneously.	
Arigliani et al., 2020<br><br>*Italy*	Case series<br><br>*Psychosocial.*	n=2<br><br>*3 years*	11 years	Clinical	**Psychiatric disorders at follow up**<br><br>*Four-day long neuropsychological evaluation.*	Case 1: Depressive conduct disorder.<br>Case 2: Anxious depressive disorder and limited oppositional behavior toward family.<br>Neither had an SM diagnosis.	
Çöpür et al., 2012<br><br>*Turkey*	Case series<br><br>*Pharmacological (citalopram or escitalopram).*	n=4<br><br>*5-9 years*	3-6 years	Clinical	SM symptoms<br><br>*Clinical evaluation and teacher reports. Cognitive assessment.*	At follow up, after citalopram treatment, 4/4 spoke in situations where they had previously stayed silent.	
Elson et al., 1965<br><br>*USA*	Case series<br><br>*Psychotherapy, in-patient treatment.*	n=4<br><br>*7-10 years*	0.5-5 years	Clinical	**SM symptoms, depression, anxiety, psychotic symptoms, sociopathic behaviour**<br><br>*Clinical, revised form of a Follow-Up Coding Manual.*	0/4 had more than mild anxiety, depression or sociopathic behaviour.<br>1/4 had symptoms of thinking disorder, but no hallucinations. 4/4 saw improvements in SM symptoms.	
Omdal & Galloway, 2008<br><br>*Norway*	Case series<br><br>*Not specified, at least psychosocial.*	n=5<br><br>*4-13 years*	1 year (the DSM-IV criteria had fulfilled at least 18 months before the	Clinical	SM symptoms<br><br>*Interviews and observation, but not specified.*	2/5 in remission. 3/5 remained selectively mute.	

			study + 1y. follow-up)				
Reed, 1963<br><br>*UK*	Case series<br><br>*Psychososical. Different psychotherapies .*	n=4<br><br>*12-13 years*	Not reported but the ages were 21 to 23 at the follow-up.	Clinical	SM symptoms<br><br>*Not reported.*	4/4 improved, but all displayed minor psychogenic abnormalities and a lack of social drive.	
Segal, 2003<br><br>*USA*	Case series<br><br>*Support in school only*	n=2<br><br>*5 years*	3 years	Clinical.<br>For mother: CBCL, Liebowitz Social Anxiety Scale, Situational Speech Scale, Physical Resemblance Questionnaire.<br>For teacher: Teacher Report Form, First Grade Readiness Tests.	SM symptoms<br><br>*Direct observation, parent and teacher interviews and assessments*	2/2 in part remission.	

Abbreviations: SM=selective mutism , CBCL= The Child Behavior Checklist

Table S4. Quality Assessment by the Quality Assessment with Diverse Studies.

Study	QuADS 1	QuADS 2	QuADS 3	QuADS 4	QuADS 5	QuADS 6	QuADS 7	QuADS 8	QuADS 9	QuADS 10	QuADS 11	QuADS 12	QuADS 13	Total points
**Arajärvi, 1965**	1	0	2	0	1	1	0	1	1	N/A	N/A	0	0	7/33
**Dogru, 2022**	3	3	2	3	2	2	3	2	2	1	3	0	2	35/39
**Kamani & Monga, 2020**	3	3	2	2	1	3	3	3	3	0	2	0	3	28/39
**Kolvin & Fundudis, 1981**	2	2	2	2	1	0	0	1	1	0	2	0	2	15/39
**Lang et al., 2016**	3	3	1	3	2	3	3	2	2	0	3	0	3	28/39
**Lowenstein, 1979**	0	1	1	1	0	0	1	1	1	N/A	N/A	0	0	6/33
**Oerbeck et al., 2018**	3	3	3	3	2	3	3	3	3	2	3	2	3	36/39
**Remschmidt et al., 2001**	3	2	3	3	2	3	3	3	3	2	3	0	3	33/39
**Sluckin et al., 1991**	3	2	1	2	1	2	3	2	1	0	2	0	1	22/39
**Steinhausen et al., 2006**	3	3	3	3	2	3	3	3	3	0	3	0	3	31/39
**Wergeland, 1979**	1	0	3	0	1	1	0	2	2	N/A	N/A	0	0	11/33
**TOTAL**	2	2	2	2	1,5	2	2	2	2	0,5	2,5	0	2	22,5
**Case series**														
**Albrigtsen et al., 2016**	2	1	3	2	1	1	2	3	0	N/A	N/A	0	2	17/33
**Arigliani et al., 2020**	1	0	1	0	0	0	0	1	0	N/A	N/A	0	0	3/33
**Çöpür et al., 2012**	2	2	1	2	0	0	1	1	0	N/A	N/A	0	0	9/33
**Elson et al., 1965**	2	2	3	2	0	2	2	2	1	N/A	N/A	0	0	16/33
**Omdal & Galloway, 2008**	3	3	1	2	0	0	0	1	1	N/A	3	0	1	15/36
**Reed, 1963**	2	2	1	2	0	1	2	1	1	N/A	N/A	0	0	12/33
**Segal, 2003**	3	2	2	2	0	2	3	2	0	N/A	N/A	0	0	16/33
**TOTAL**	2	1,5	1,5	1,5	0	1	1,5	1,5	0,5	0	3	0	0,5	12
